# New Assays to Characterise Growth-Related Phenotypes of *Plasmodium falciparum* Reveal Variation in Density-Dependent Growth Inhibition between Parasite Lines

**DOI:** 10.1371/journal.pone.0165358

**Published:** 2016-10-25

**Authors:** Núria Rovira-Graells, Sara Aguilera-Simón, Elisabet Tintó-Font, Alfred Cortés

**Affiliations:** 1 ISGlobal, Barcelona Ctr. Int. Health Res. (CRESIB), Hospital Clínic - Universitat de Barcelona, Barcelona, Catalonia, Spain; 2 ICREA, Barcelona, Catalonia, Spain; Liverpool School of Tropical Medicine, UNITED KINGDOM

## Abstract

The growth phenotype of asexual blood stage malaria parasites can influence their virulence and also their ability to survive and achieve transmission to the next host, but there are few methods available to characterise parasite growth parameters in detail. We developed a new assay to measure growth rates at different starting parasitaemias in a 96-well format and applied it to characterise the growth of *Plasmodium falciparum* lines 3D7-A and 3D7-B, previously shown to have different invasion rates and to use different invasion pathways. Using this simple and accurate assay we found that 3D7-B is more sensitive to high initial parasitaemia than 3D7-A. This result indicates that different parasite lines show variation in their levels of density-dependent growth inhibition. We also developed a new assay to compare the duration of the asexual blood cycle between different parasite lines. The assay is based on the tight synchronisation of cultures to a 1 h parasite age window and the subsequent monitoring of schizont bursting and formation of new rings by flow cytometry. Using this assay we observed differences in the duration of the asexual blood cycle between parasite lines 3D7 and HB3. These two new assays will be useful to characterise variation in growth-related parameters and to identify growth phenotypes associated with the targeted deletion of specific genes or with particular genomic, transcriptomic or proteomic patterns. Furthermore, the identification of density-dependent growth inhibition as an intrinsic parasite property that varies between parasite lines expands the repertoire of measurable growth-related phenotypic traits that have the potential to influence the outcome of a malarial blood infection.

## Introduction

*Plasmodium falciparum* is responsible for the majority of malaria deaths and severe disease cases, and consequently is the subject of intensive investigations. Recent research advances have raised hopes that a better understanding of the basic biology of this organism may contribute to the design and development of new public health tools that will assist in the fight against malaria. To name just a few landmark research advances from the last fifteen years, the complete genome of *P*. *falciparum* has been sequenced [1], enabling the characterisation of the parasite at the “omics” level [2,3,4,5,6,7,8], and genetic manipulation technology has advanced to a point that efficient knock out, conditional depletion, forward genetic screening or targeted mutagenesis are becoming routine [9].

In contrast to these major advances, the methods for parasite phenotypic characterisation have not progressed at the same pace. The repertoire of easy methods for phenotypic characterisation is limited, and consequently the number of phenotypic traits that are typically determined for a parasite line is clearly insufficient to cope with the wealth of new “omics” datasets or new transgenic lines that are constantly being generated. Specific genetic, transcriptomic, epigenomic, proteomic or phosphoproteomic patterns may be associated with specific phenotypes, but such associations will not be detected unless these phenotypes are routinely characterised. Likewise, the genetic modification of specific loci may be associated with phenotypes that currently are not determined as part of the regular characterisation of new transgenic lines. Of note, the only growth-related phenotypic trait that is characterised for many transgenic parasite lines is the growth rate.

The characterisation of the growth phenotype of asexual blood stage parasites, which are responsible for all clinical symptoms of malaria, is particularly important. During the intraerythrocytic cycle, parasites develop and replicate inside the erythrocytes for about 48 h and are then released to the circulation, where they quickly invade new erythrocytes to start the next replication cycle. The growth parameters of a parasite line, such as the duration of the asexual blood cycle or the increase in parasitaemia from one cycle to the next (growth rate), are major intrinsic properties of a parasite line that may influence its virulence [10] and also its ability to survive and achieve transmission, especially in the context of a multiple infection in which several parasite clones coexist and compete for limited resources [11]. The growth dynamics of a parasite line also depends on the trade-off between asexual blood multiplication and differentiation into sexual precursors called gametocytes, which are necessary for transmission to a mosquito and subsequently to another human host. Importantly, the investment on gametocyte production varies between different parasite lines [12,13,14]. In addition to these parasite intrinsic factors that can be measured *in vitro* under culture conditions, host factors such as innate and acquired immune responses also influence the growth dynamics of a malaria infection in the human blood [15].

Over a decade ago, we described two stocks of the *P*. *falciparum* clonal line 3D7, named 3D7-A and 3D7-B (distinct from 3D7A and 3D7B described in the first malaria genetic cross [16]), which use different invasion pathways and have different invasion rates [17]. Using regular erythrocyte invasion assays, in which the number of new rings generated per purified schizont is determined, 3D7-A showed higher invasion rates than 3D7-B. Of note, 3D7-A is unique in its invasive abilities because it is the only parasite line reported to date that can invade efficiently erythrocytes from individuals with the malaria-protective genetic trait South-East Asian ovalocytosis (SAO) [17] and also normal erythrocytes sequentially treated with neuraminidase and trypsin [17,18]. The 3D7-A and 3D7-B stocks of 3D7 were instrumental to investigate epigenetic variation in *P*. *falciparum* [8,18].

During routine culture of 3D7-A and 3D7-B, we noticed that 3D7-A exhibits a higher growth rate, consistent with its previously reported higher invasion rate [17]. For regular culture maintenance, we assume an eight-fold increase in parasitaemia from one cycle to the next for 3D7-A, and about six-fold for 3D7-B. We also observed that the difference in growth rates between the two parasite lines is more pronounced when they are cultured at high parasitaemia. To formally assess this latter observation, we developed a new assay to compare growth rates at different starting parasitaemias. We optimised the method for a 96-well plate format and used it to characterise density-dependent growth inhibition in 3D7-A and 3D7-B, and also in other parasite lines. Furthermore, we developed an assay to accurately measure the length of the asexual blood cycle and to characterise the growth kinetics. Our results revealed variation for all growth-related phenotypic traits analysed.

## Results

### Growth rates of 3D7-A and 3D7-B at different starting parasitaemias in Petri dishes

**Fig 1:**
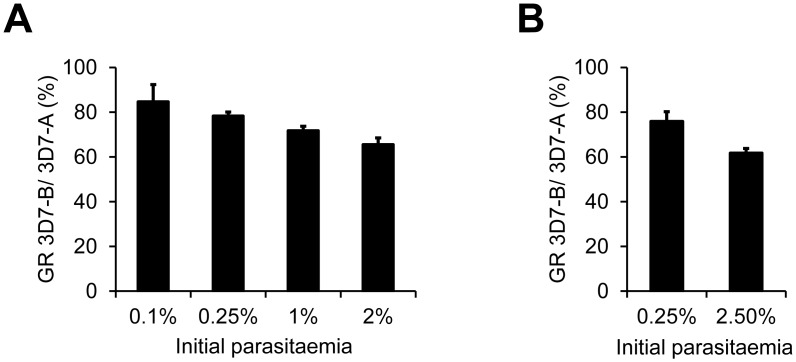
Growth rates of 3D7-A and 3D7-B at different initial parasitaemias measured in Petri dish assays. (A) Growth rates of the two parasite lines were measured at four different starting parasitaemias. Values are the growth rate (GR) of 3D7-B relative to 3D7-A at each initial parasitaemia, expressed as percentage. Values are the average of four independent biological replicates, with S.E.M. The decrease of the growth rate of 3D7-B relative to 3D7-A with increasing initial parasitaemia was statistically significant (*P*<0.05) using linear regression analysis. (B) Same as in panel A, but in a second set of experiments in which growth rates were measured only at two different starting parasitaemias. Values are the average of seven independent biological replicates, with S.E.M. The growth rate of 3D7-B relative to 3D7-A was significantly different between the two starting parasitaemias (*P*<0.05 using a two-tailed paired *t*-test).

In an initial set of exploratory experiments, we measured the growth rate of 3D7-A and 3D7-B cultures at starting parasitaemias ranging from 0.1 to 2% (Fig 1A). In brief, the parasitaemia of synchronised cultures at the ring stage was adjusted to the desired starting parasitaemias by dilution with uninfected erythrocytes. To determine the growth rate, parasitaemias were measured ~55 h later when all parasites had completed a full asexual multiplication cycle and reached the ring / early trophozoite stage of the next cycle. We used flow cytometry to determine parasitaemias accurately. The growth rate was calculated as the final parasitaemia divided by the initial parasitaemia. Hence, progression along the full asexual blood cycle is assessed in these growth assays, in contrast to invasion assays in which only progression from the schizont to the ring stage is analysed. In four independent experiments, 3D7-A consistently showed higher growth rates than 3D7-B at all starting parasitaemias. However, differences were more marked in cultures at high parasitaemia. The growth of 3D7-B was ~80% the growth of 3D7-A at 0.1% or 0.25% initial parasitaemia, but only ~65% in cultures at 2% initial parasitaemia (Fig 1A).

Based on these results, we performed a second round of experiments using only 0.25% and 2.5% starting parasitaemias (Fig 1B). These experiments confirmed that 3D7-B is more sensitive to high parasite density than 3D7-A, such that at the lower starting parasitaemia it grows ~75% as much as 3D7-A but at the higher starting parasitaemia it only grows ~60% as much as 3D7-A. In these experiments we used both old (stored for >3 weeks) and fresh (stored for 1–2 weeks) erythrocytes, but we did not observe differences between the two conditions. Altogether, these assays confirmed our previous observations during routine culture that density-dependent growth inhibition is stronger in 3D7-B than in 3D7-A.

### A 96-well plate assay to measure growth rates at different starting parasitaemias

We designed a simple and accurate new assay to measure growth rates at different starting parasitaemias in 96-well plates. In this assay, which can be used for routine phenotypic characterisation of parasite lines, the growth rate of each line is tested at six different initial parasitaemias ranging from 0.08% to 2.5%. Serial 1 in 2 dilutions are used to generate wells with progressively lower starting parasitaemias. We observed some variability between experiments in the growth rates measured, probably as a consequence of using blood from different donors; hence, for optimal accuracy, the parasite lines compared need to be analysed in parallel.

**Fig 2:**
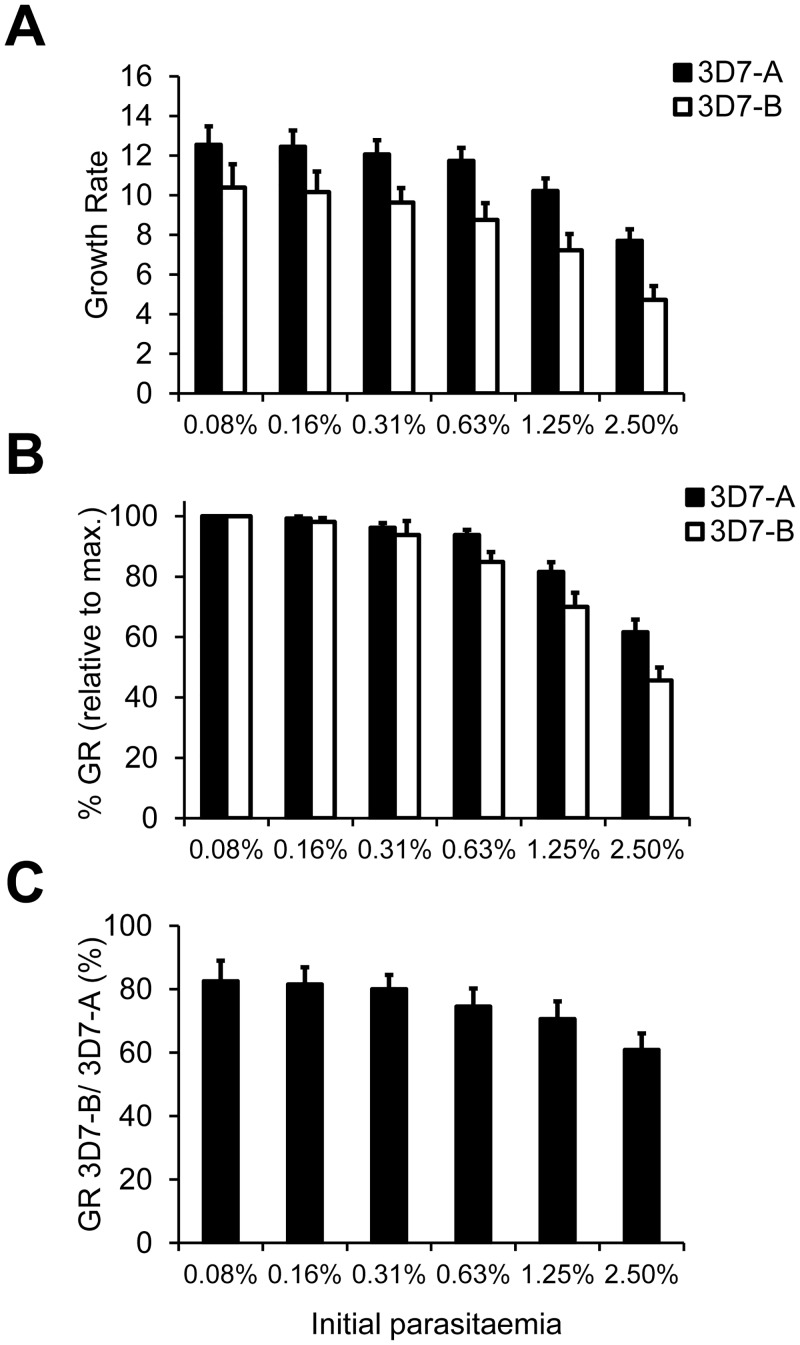
Growth rates of 3D7-A and 3D7-B at different initial parasitaemias measured in 96-well plate assays. (A) Growth rates of 3D7-A and 3D7-B in cultures at different starting parasitaemias obtained by serial dilutions in 96-well plates. (B) Growth rates (GR) at different starting parasitaemias relative to the growth rate at the lowest starting parasitaemia (typically the maximum growth rate) for each parasite line. (C) Growth rate of 3D7-B relative to 3D7-A at different starting parasitaemias, expressed as percentage. The decrease of the growth rate of 3D7-B relative to 3D7-A with increasing initial parasitaemia was statistically significant (*P*<0.05) using linear regression analysis. In all panels, values are the average of three independent experiments (biological replicates), each performed in triplicate wells, with S.E.M.

Using this assay, we confirmed the conclusions of the experiments performed in Petri dishes. The maximum growth rate, typically observed in wells at the lowest starting parasitaemia, was about twelve for 3D7-A and about ten for 3D7-B. For both parasite lines growth rates progressively decreased with higher starting parasitaemia, but this was more pronounced for 3D7-B (Fig 2A). The different levels of density-dependent inhibition of growth between 3D7-A and 3D7-B are more apparent by representing growth rates at each starting parasitaemia as the percentage of the growth rate of the same parasite line at the lowest starting parasitaemia (typically the maximum growth rate). In three independent experiments 3D7-B showed a clearly higher sensitivity to high initial parasitaemia than 3D7-A, such that the growth of 3D7-B at the highest starting parasitaemia was only about 45% of its maximum growth rate, whereas in the case of 3D7-A it was over 60% (Fig 2B). The growth rate of 3D7-B relative to 3D7-A was ~80% in wells at low starting parasitaemia but progressively declined to only ~60% in wells at the highest starting parasitaemia (Fig 2C).

**Fig 3:**
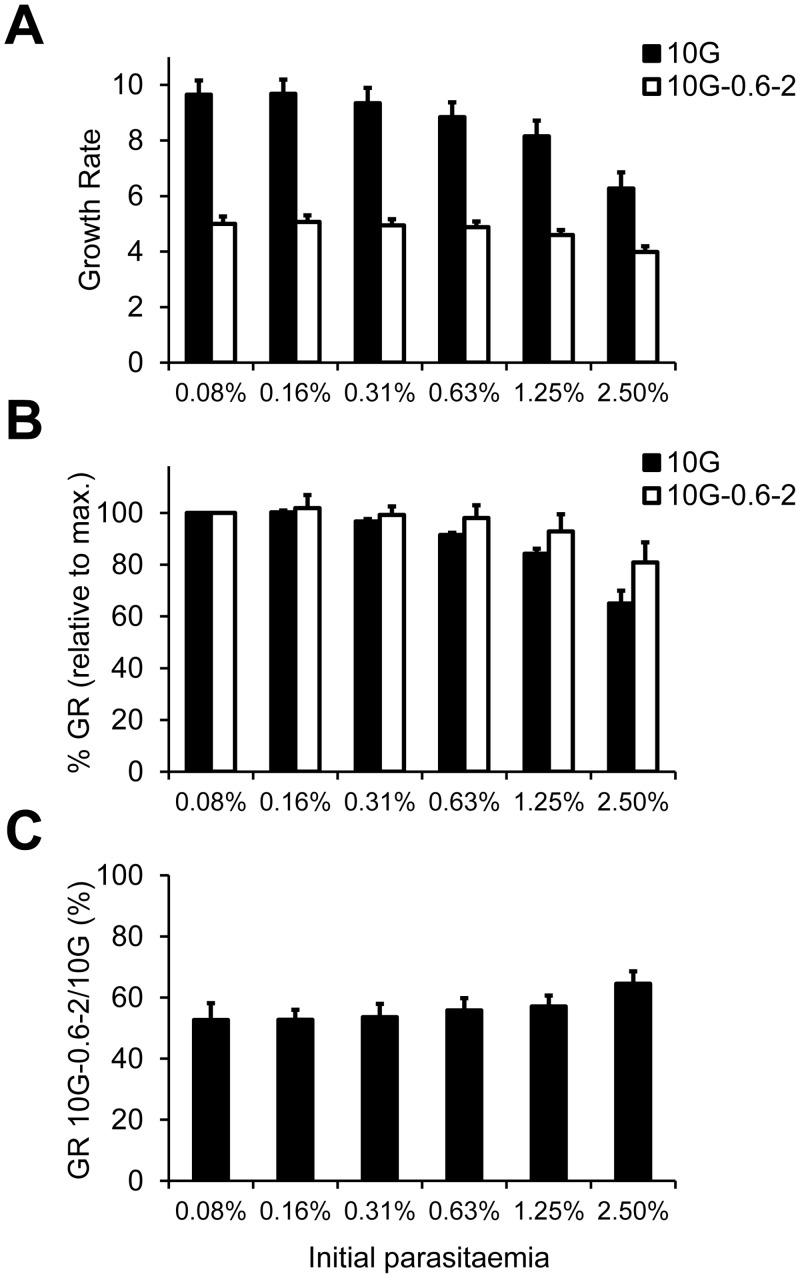
Growth rates of 10G and 10G-0.6–2 at different initial parasitaemias measured in 96-well plate assays. (A) Growth rates of 10G and 10G-0.6–2 in cultures at different starting parasitaemias obtained by serial dilutions in 96-well plates. (B) Growth rates (GR) at different starting parasitaemias relative to the growth rate at the lowest starting parasitaemia (typically the maximum growth rate) for each parasite line. (C) Growth rate of 10G-0.6–2 relative to 10G at different starting parasitaemias, expressed as percentage. The increase of the growth rate of 10G-0.6–2 relative to 10G with increasing initial parasitaemia was statistically significant (*P*<0.05) using linear regression analysis. In all panels, values are the average of four independent experiments (biological replicates), each performed in duplicate wells, with S.E.M.

In summary, we have established a simple and accurate assay to compare the growth rates of different parasite lines at different starting parasitaemias, which revealed that different lines show different levels of sensitivity to high parasitaemia. To further demonstrate the utility of this assay, we used it to analyse the density-dependence of a parasite line of interest that we recently generated by selecting wild type parasite cultures with the toxic compound blasticidin S [19]. This parasite line, termed 10G-0.6–2, acquired resistance to blasticidin S by silencing the expression of *clag3* genes, and displays markedly reduced growth rates compared to the 10G parental line from which it was derived [19]. At low starting parasitaemias, 10G-0.6–2 grows at about 50% the rate of 10G (Fig 3A and 3C). Surprisingly, in spite of its predicted limited capacity to acquire sufficient nutrients from the medium [19,20], the growth rate of 10G-0.6–2 shows little variation with initial parasite density, such that at the highest initial parasitaemia this parasite line grows at over 80% the rate at the lowest parasitaemia (Fig 3B). Thus, 10G-0.6–2 shows a lower density-dependent inhibition of growth than unselected 10G (Fig 3A–3C), which at the highest parasitaemia only grows at just over 60% the rate at the lowest parasitaemia. At the highest starting parasitaemia (2.5%) the growth of 10G-0.6–2 relative to 10G is over 60% (Fig 3C).

### The number of merozoites per schizont is similar between 3D7-A and 3D7-B

**Fig 4:**
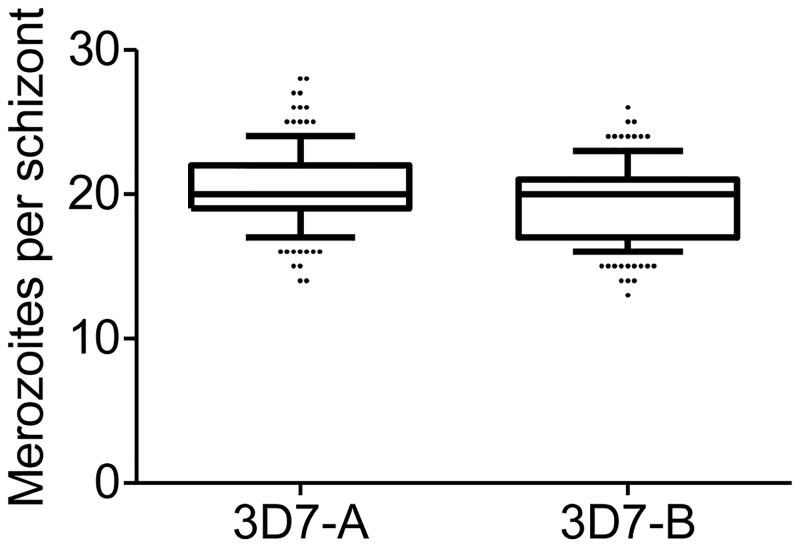
Number of merozoites per schizont in 3D7-A and 3D7-B. The number of merozoites was counted in 160 (3D7-A) or 165 (3D7-B) fully mature, segmented schizonts from three independent preparations of tightly-synchronised cultures. Boxes represent median, 25^th^ percentile and 75^th^ percentile, whereas whiskers are 10^th^ and 90^th^ percentiles. The mean number of merozoites per schizont was significantly different between 3D7-A and 3D7-B (*P*<0.05) using a two-tailed *t*-test.

The lower growth rates of 3D7-B compared to 3D7-A may be explained by either 3D7-B producing less merozoites per schizont, or 3D7-B merozoites invading erythrocytes less efficiently. To distinguish between these two possibilities, we counted the number of merozoites per fully mature schizont in the two parasite lines, using tightly synchronised cultures at 46–47 or 48–49 h post-invasion (Fig 4). The median number of merozoites per schizont was 20 in both parasite lines, whereas the mean was 20.44 for 3D7-A and 19.29 for 3D7-B. Although this small difference was statistically significant (*P*<0.05 using a *t*-test), the very small increase (~6%) in the mean number of merozoites per schizont in 3D7-A is insufficient to account for the highly increased growth rates of this line (~20% higher even at low starting parasitaemia, Figs 1 and 2C). Thus, we conclude that the higher growth rate of 3D7-A compared to 3D7-B is mainly attributable to more efficient merozoite invasion, consistent with the reported ability of this parasite line to use additional invasion pathways [17,18], rather than to an increased number of merozoites per schizont.

### An assay to compare the duration of the asexual blood cycle between parasite lines

**Fig 5:**
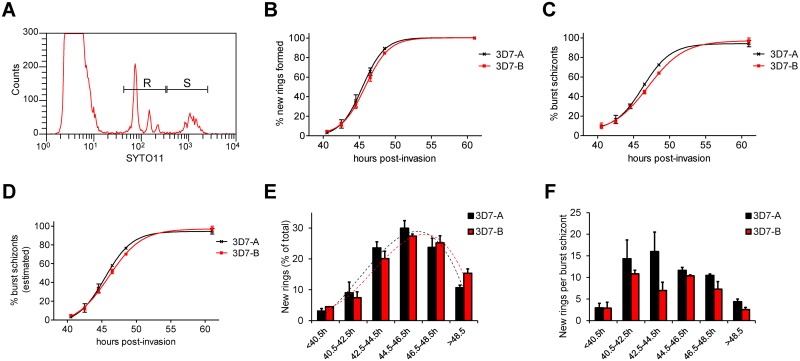
Duration of the asexual blood cycle in 3D7-A and 3D7-B. (A) Representative example of a flow cytometry analysis showing the position of peaks of ring-infected erythrocytes (R window, including single, double, triple and quadruple ring-infected erythrocytes) and schizont-infected erythrocytes (S window). (B) Cumulative number of new rings formed at different time points, expressed as the proportion (%) of the total number of rings (determined at ≥60 h, when reinvasion was complete). Values in the x-axis correspond to average culture age in h post-invasion. Data are fitted to a sigmoidal dose-response curve. (C) Cumulative proportion (%) of burst schizonts at different time points. The proportion of burst schizonts was calculated from the schizont parasitaemia as described in the Materials and Methods section. (D) Cumulative proportion (%) of burst schizonts at different time points, estimated from the relative ring and schizont parasitaemia, as described in the Materials and Methods. (E) Proportion (%) of new rings that were formed at each time interval. Values in the x-axis are time intervals defined by the average culture age (in h post-invasion) at the beginning and at the end of the interval. The proportion of new rings formed during each time interval is calculated as the increase in ring parasitaemia during the interval divided by the total ring parasitaemia at the end of the assay. The trend line is a polynomial of degree three (dotted lines). (F) Average number of new rings formed per burst schizont at different intervals of cycle progression. The number of new rings per schizont is calculated as Δ*Pr*/(-Δ*Ps*), where Δ*Pr* and -Δ*Ps* are the increase in ring parasitaemia and the decrease in schizont parasitaemia, respectively, during the interval. In all panels, values are the average of two independent biological replicates, with S.E.M.

We developed a new assay to measure another fundamental growth parameter of malaria parasites: the duration of the asexual blood cycle. In brief, we synchronised cultures to a 1 h age window by sequential Percoll purification and sorbitol lysis (0–1 h post-invasion culture), and towards the end of the asexual cycle we followed schizont disappearance (as a consequence of bursting) and formation of new rings by flow cytometry (Fig 5A). With this approach, we found that the duration of the asexual blood cycle is very similar between 3D7-A and 3D7-B, according to both the formation of new rings and the decrease in the number of schizonts (Fig 5B and 5C and Table 1). Because measurements of the very low schizont parasitaemias observed at the latest time points have limited accuracy, we also estimated the proportion of burst schizonts at a given time point from the relative abundance of rings and schizonts (see Materials and Methods for details). The progression of schizont bursting estimated in this way was also very similar between the two parasite lines (Fig 5D).

**Table 1:** Duration of the asexual blood cycle in different *P*. *falciparum* lines.

% of new rings formed	3D7-A vs 3D7-B		3D7-A vs HB3A	
	3D7-A	3D7-B	3D7-A	HB3A
20%	43.3	43.5	43.3	44.9
50%	45.4	45.8	45.9	47.7
80%	47.5	48.1	48.8	50.7

Time (in h post-invasion) elapsed until the indicated proportion of the total of new rings was formed. Values were obtained by interpolating sigmoidal dose-response curves (variable slope) generated with data from two independent experiments.

We also calculated the proportion of the new rings formed at each time interval, which revealed that in both 3D7-A and 3D7-B the peak of new rings formation was at ~45 h post-invasion (Fig 5E). Although the cultures were synchronised to a 1 h window, bursting and formation of new rings occurred over a period of >8 h. Next we calculated the average number of new rings formed per burst schizont for each of the time intervals (Fig 5F). We observed high variability between experiments for this parameter, which is probably attributable to the different absolute growth rates observed in separate experiments (related with the use of blood from different donors) and to the limited accuracy of measuring small decreases in schizont parasitaemia across short periods of time. In spite of these limitations, this analysis revealed that fifteen rings or more can be formed per burst schizont during intervals of peak efficiency of invasion. In contrast, at the first and the last time intervals analysed, the number of new rings per burst schizont was much lower. The low values before 40.5 h are likely explained by parasites that die before completing schizont maturation and bursting, whereas the low values after 48.5 h reflect a decreased ability of the merozoites arising from the latest schizonts that burst to invade new erythrocytes efficiently. At some specific intervals, the number of rings per burst schizont was higher for 3D7-A than for 3D7-B. This was expected because while the kinetics of cycle progression is similar between the two lines (Fig 5B–5E), the absolute number of rings formed from each schizont is directly linked to the growth rate, which is higher in 3D7-A (Figs 1 and 2). Determining whether differences between the two lines in the number of rings per burst schizont occur specifically at ~40–45 h post-invasion will require additional experimental approaches.

**Fig 6:**
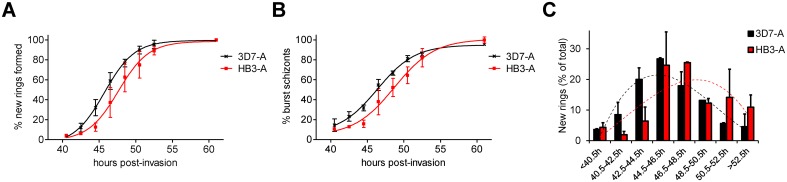
Duration of the asexual blood cycle in 3D7-A and HB3A. The duration of the asexual blood cycle was compared between the 3D7-A and HB3A parasite lines. Experiments in panels (A), (B) and (C) are analogous to experiments in panels B, C and E of Fig 5, respectively. Results are the average of two independent biological replicates, with S.E.M.

We previously proposed that the duration of the asexual blood cycle may be slightly shorter in 3D7-A than in 3D7-B [18], and in fact a very small difference (<1 h) was observed in this direction for all the parameters measured (Fig 5B–5E and Table 1). However, the accuracy of our assay is not sufficient to determine such small differences with confidence. To test whether our assay is able to identify variation in the duration of the cycle when differences are more pronounced, we used it to compare two parasite lines of different genetic background, 3D7-A and HB3A. We observed a ~2 h difference in the time necessary to complete the asexual blood cycle between the two parasite lines. While 50% of the new rings were generated in less than 46 h in 3D7-A, in HB3A this required almost 48 h (Fig 6A and Table 1). The time elapsed until 20% or 80% of the new rings were formed also differed by almost 2 h between the two parasite lines (Table 1). The kinetics of schizonts disappearance confirmed this difference in the speed of asexual cycle progression (Fig 6B). Last, the analysis of the proportion of rings formed at each time interval also revealed that the peak of new rings formation occurred earlier in 3D7-A than in HB3A (Fig 6C). Altogether, these results demonstrate that our method is suitable for the identification of differences in the speed of life cycle progression between parasite lines.

## Discussion

Here we describe new assays for the characterisation of growth-related phenotypic traits in *P*. *falciparum*, including a method to determine the density-dependence of growth rates. We observed variation between parasite lines for all of the traits analysed, which in addition to density-dependent growth inhibition included maximum growth rate, duration of the asexual blood cycle, and number of merozoites per segmented schizont. The establishment of robust assays to characterise these phenotypes opens the way to study the molecular basis of this variation. Considering that differences in some of the intrinsic growth parameters were observed between parasite lines of the same genetic background (3D7-A and 3D7-B), it is likely that they have an epigenetic basis and are related with differential expression of clonally variant genes [8]. However, we cannot rule out the possibility that these phenotypic differences are explained by unknown genetic alterations that may have occurred during normal growth under culture conditions. In any case, the phenotypic variation observed for these traits has an important adaptive potential, as it provides the grounds for dynamic natural selection of parasites with characteristics that confer increased fitness upon changes in the host conditions.

Parasites that have a very high intrinsic growth rate or a comparatively short asexual blood cycle are expected to have a competitive advantage in the context of a patient infected with multiple parasite clones [11], which is a very common situation in malaria endemic areas [21]. Furthermore, traits associated with fast growth can favour transmissibility and parasite survival in human hosts with strong acquired immune responses. However, fast growth may also be detrimental for the parasite because it carries the risk of killing the human host, which would result in parasites death. The trade-off between preventing excess host damage and growing sufficiently fast to avoid being outcompeted by other parasite clones or eliminated by the host immune system needs to be tightly regulated, making growth-related phenotypes critically important for parasite survival. However, the plasticity of asexual growth traits remains poorly characterised [22].

We found that in addition to exhibiting different maximum growth rates, different parasite lines vary in their sensitivity to high parasite density, demonstrating that the level of density-dependent growth inhibition is an intrinsic property of a parasite line. Density-dependent inhibition of growth has been proposed to occur also during natural infections, even across species [23]. Growth restriction at high parasitaemia is intuitively a favourable trait for parasites, to prevent fast growth that may result in host death when the parasite biomass is already high. In fact, the optimal growth strategy for a malaria parasite population may involve fast multiplication rates at the beginning of a blood infection followed by growth restriction regulated by quorum-sensing mechanisms when parasitaemia is high. We hypothesise that the different levels of density-dependent growth inhibition observed between two lines of 3D7 genetic background, 3D7-A and 3D7-B, may reflect a different use of quorum-sensing mechanisms. Extracellular vesicles containing parasite-derived materials, which were recently described for *Plasmodium* spp. [24,25,26], may be responsible for cell-cell communication and quorum-sensing in malaria. In this context, 3D7-A and 3D7-B may differ in their ability to either produce or receive these vesicles. Our assay to easily and accurately compare the level of density-dependent growth inhibition of different parasite lines will be useful to investigate the mechanism underlying this phenotypic trait and to test the hypothesis that it is related to quorum-sensing and extracellular vesicles.

We also used this assay to characterise the density-dependence of the growth rate of 10G-0.6–2, a parasite line with reduced growth as a consequence of silencing the expression of *clag3* genes after selection with blasticidin S [19]. These genes are necessary for efficient nutrient uptake [20,27]. The 10G-0.6–2 parasite line showed a low level of growth inhibition at high parasitaemia compared to the unselected 10G line from which it was derived. Considering that for a given initial parasitaemia the density of new rings is lower in 10G-0.6–2 than in other parasite lines, this result may suggest that inhibition of growth by high parasite densities mainly depends on the parasitaemia of new rings formed, rather than on the parasitaemia of schizonts before invasion. However, the absence of CLAG3 expression likely results in multiple alterations at different stages of the life cycle in the 10G-0.6–2 line, so this hypothesis requires confirmation. Future research will need to establish the precise mechanism regulating density-dependent growth inhibition and the involvement of parasites at different stages of development.

We also developed a method to accurately measure the duration of the asexual blood cycle, which revealed that the cycle length in parasites of 3D7 genetic background is on average ~46 h, and about 2 h longer in HB3. Variation in cycle length duration between parasite lines of different genetic background has been previously described and proposed to be under genetic control [28,29,30]. Other methods have been previously described to measure the duration of the asexual blood cycle. One study provided a detailed structural description of schizont development, bursting and reinvasion using cultures synchronised to a ~1.5 h window, and identified differences in the duration of the asexual cycle between two parasite lines of different genetic background [28]. This study also concluded that in a single cycle the window of culture synchrony widens markedly, similar to our observations: we found substantial schizont bursting over a period of >8 h in cultures that had been previously synchronised to only a 1 h age window. However, the method developed by Margos et al. involved the use of chemical inhibitors to synchronise the cultures, and relied on microscopy to estimate the proportion of parasites at different stages, which is less accurate than flow cytometry. Another method to measure the duration of the asexual cycle is based on estimating the time between two peaks of schizonts [31,32]. While this method was also suitable to reliably measure the duration of the life cycle and to compare the speed of cycle progression between different parasite lines, it required frequent sampling along a 56–90 h experiment. In contrast, in our protocol frequent sample collection is restricted to 8–12 h. Nevertheless, we are aware that this is a labour-intensive protocol, and it requires growing relatively large volumes of parasite cultures to obtain a high enough parasitaemia after synchronisation to a 1 h age window. While we do not envisage the use of this method in high-throughput experiments, the protocol described here can be valuable for the phenotypic characterisation of important parasite lines with defined genetic or epigenetic alterations (e.g. transgenic lines) that can potentially affect the duration of the life cycle.

Altogether, we provide a description of much needed new methods for the detailed characterisation of growth phenotypes in *P*. *falciparum*, and we used these methods to characterise the growth of several parasite lines including two lines of 3D7 genetic background with markedly different phenotypes. These methods will be useful for the characterisation of plasticity and variation in asexual growth traits. Of special interest was the identification of variation in the levels of density-dependent growth inhibition. Further characterisation of this trait may reveal important survival and adaptive mechanisms of malaria parasites.

## Materials and Methods

### Parasites and flow cytometry

The parasite lines used in this study have been previously described: 3D7-A and 3D7-B [17], 10G [33], 10G-0.6–2 [19] and HB3A [16]. Parasites were cultured in B+ erythrocytes in a low oxygen (2%) atmosphere under standard conditions with Albumax II and no human serum. We used standard sorbitol lysis for regular synchronisation of all parasite lines except for 10G-0.6–2, which was synchronised with L-Proline instead of sorbitol as previously described [34]. This parasite line was regularly cultured under blasticidin S pressure to maintain its characteristics [19].

Flow cytometry determination of parasitaemia was performed as previously described [35], using a FACScalibur flow cytometer (Becton Dickinson) and SYTO 11 to stain parasite nucleic acids. While other fluorescent dyes such as ethidium bromide show a limited ability to stain early ring stage parasites for flow cytometry analysis [36], SYTO 11 efficiently stains young rings even in 0–1 h post-invasion cultures (S1 Fig). The ability to detect very young rings in our flow cytometry assay is important in order to rule out the possibility that in some parasite lines a subset of new rings fail to develop, rather than merozoites invading erythrocytes less efficiently. The background signal that was scored as parasitaemia in uninfected erythrocytes preparations was determined in each experiment (typically ~0.03%) and subtracted from other parasitaemia measurements. Uninfected erythrocytes were maintained at 37°C for ~48 h before they were used in the growth assays because we observed that when used directly after storage at 4°C the background SYTO 11 signal is higher. For flow cytometry quantification of rings and schizonts in cultures containing a mixture of parasites at both stages (Fig 5A), we manually defined windows of SYTO 11 signal corresponding to one to approximately four nuclei (rings, including erythrocytes infected with multiple rings) and to more than four nuclei (schizonts).

### Growth assays

Parasitaemia was measured by flow cytometry after synchronising cultures to the ring stage, and adjusted to the highest starting parasitaemia by dilution with a suspension of uninfected erythrocytes (in complete culture medium) at the same haematocrit as the cultures. We confirmed that the haematocrit was the same between the cultures and the uninfected erythrocytes diluent by comparing pellet volumes after centrifugation. If differences were observed, the haematocrit was adjusted between the two preparations. The parasitaemia of the adjusted preparations was confirmed by flow cytometry. We prepared dilutions to all the other desired starting parasitaemias using the same uninfected erythrocytes diluent. This was done in 15 ml tubes for assays in Petri dishes or directly in a 96-well plate (in duplicate or triplicate wells) with serial 1 in 2 dilutions using a multichannel pipette. In several cases we validated the parasitaemia of all the dilutions and they were found to be approximately as expected. However, since flow cytometry measurements of low parasitaemias (<0.2%) have limited accuracy, for all the data analyses we used the initial parasitaemias calculated from the highest initial parasitaemia (measured by flow cytometry) divided by the dilution factor. Cultures were left undisturbed for >50 h until reinvasion was complete (as determined by microscopy examination) and then the parasitaemia of each dish or well was measured by flow cytometry. Growth rates were calculated as the final parasitaemia divided by the initial parasitaemia. For assays with the parasite line 10G-0.6–2, blasticidin S was removed immediately before starting the assay.

### Synchronisation of cultures to a 1 h window and measurement of the duration of the asexual growth cycle

Cultures containing abundant schizonts (~5% schizont parasitaemia) were synchronised to a 1 h window by Percoll purification of schizonts followed by sorbitol lysis 1 h later. Since late stage parasites (trophozoites and schizonts) were very abundant 1 h after Percoll purification, we modified our standard sorbitol lysis protocol to ensure that they were completely removed. In brief, the volume of sorbitol was increased from 5–10 to 30 pellet volumes, the time of incubation with sorbitol was increased from 7 to 10 minutes, and we performed two washes after the incubation with sorbitol instead of one. Parasitaemia was determined by flow cytometry and adjusted to 1.25% by dilution with uninfected erythrocytes as described above. Cultures were split between several identical dishes (0–1 h post-invasion cultures) that were left undisturbed until they were harvested at different time points for flow cytometry measurement of ring and schizont parasitaemias. Initial parasitaemia was determined at ~20 h post-invasion, a time point at which bursting and reinvasion has not started yet. The value of schizont parasitaemia at ~20 h post-invasion, which likely corresponds to dead schizonts that were not removed by sorbitol lysis, was subtracted from subsequent schizont parasitaemia measurements. Ring and schizont parasitaemias were determined at two hours intervals during the period in which schizont bursting and reinvasion occurred, and at ≥60 h post-invasion when all viable schizonts had burst.

The proportion of burst schizonts at a given time point was estimated from the experimentally determined schizont parasitaemia or from the relative ring and schizont parasitaemias. For the former approach, the proportion of burst schizonts was calculated as 100 –[(*Ps*/*Pi*) x 100], where *Ps* is the schizont parasitaemia at a given time point and *Pi* is the initial parasitaemia determined at about 20 h post-invasion. For the latter approach, the proportion of burst schizonts was calculated using the formula: 100 x (*Pr*/*GR*)/[(*Pr*/*GR*)+*Ps*], where *Pr* and *Ps* are the parasitaemia of rings and schizonts at a given time point, respectively, and *GR* is the growth rate determined in the same experiment by dividing the final ring parasitaemia at ≥60 h post-invasion by the initial parasitaemia. In this way, (*Pr*/*GR*) is an estimation of the number of schizonts that have burst at a given time point, whereas [(*Pr*/*GR*)+*Ps*] is an estimation of the initial total number of schizonts. Data was fitted to a sigmoidal dose-response curve with variable slope using GraphPad Prism, and the time to generate 20%, 50% or 80% of new rings for each parasite line was interpolated from the curves.

### Determination of the number of merozoites per mature schizont

Giemsa-stained smears from parasite cultures at ~1.25% parasitaemia synchronised to a 1 h age window were analysed by light microscopy at 46–47 or 48–49 h post-invasion. We obtained pictures from multiple high magnification fields (1000x) of each slide and counted the number of merozoites per fully mature schizont on a computer screen. We excluded schizonts with non-defined or overlapping merozoites, as well as those with multiple hemozoin pigment signals that may indicate infection of the same erythrocyte by more than one parasite.

## Supporting Information

S1 Fig Detection of very early rings by flow cytometry. (PDF)
