# Supplementary material for: New Assays to Characterise Growth-Related Phenotypes of Plasmodium falciparum Reveal Variation in Density-Dependent Growth Inhibition between Parasite Lines
Source: PLoS One. 2016 Oct 25;11(10):e0165358. doi: 10.1371/journal.pone.0165358 (PMC5079629; doi:10.1371/journal.pone.0165358)
Supplement: S1 Fig — (PDF) [file pone.0165358.s001.pdf]

SUPPORTING INFORMATION

New Assays to Characterise Growth-Related Phenotypes of *Plasmodium falciparum* Reveal Variation in Density-Dependent Growth Inhibition between Parasite Lines

Núria Rovira-Graells, Sara Aguilera-Simón, Elisabet Tintó-Font & Alfred Cortés

A

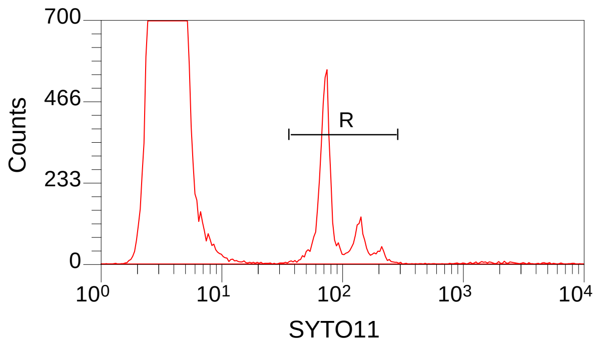

B

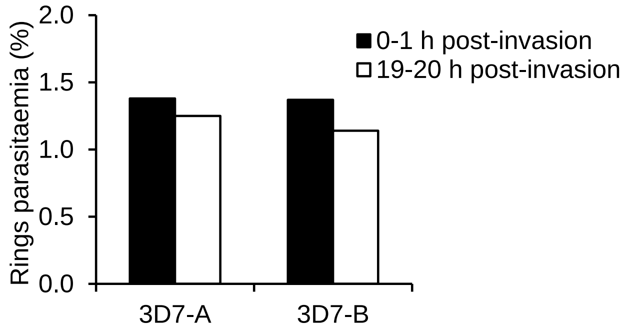

C

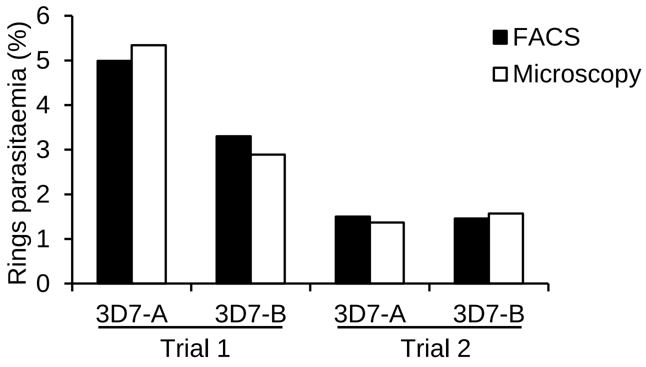

S1 Fig. Detection of very early rings by flow cytometry. (A) Representative example of a flow cytometry analysis of 0-1 h post-invasion cultures. R indicates the position of peaks corresponding to erythrocytes infected with one or more ring-stage parasites. (B) Comparison of parasitaemia measured by flow cytometry in the same cultures at 0-1 h post-invasion and 19 h later. (C) Comparison of parasitaemia in a 0-1 h post-invasion culture measured by flow cytometry (FACS) or by microscopy analysis of Giemsa-stained smears.

These results demonstrate that our flow cytometry assay, which uses the SYTO 11 fluorescent dye to stain parasite nucleic acids, accurately detects very early ring stage parasites.
